# A Survey of the Brazilian *Dicranocentrus* Schött (Collembola, Orchesellidae, Heteromurini) with the Description of a New Species and Notes on the Genus

**DOI:** 10.3390/insects11100709

**Published:** 2020-10-16

**Authors:** Bruno C. Bellini, Nikolas G. Cipola, Orquianne J. R. Siqueira

**Affiliations:** 1 Laboratório de Collembola, Departamento de Botânica e Zoologia, Centro de Biociências, Universidade Federal do Rio Grande do Norte—UFRN, BR 101, Lagoa Nova, Campus Universitário, Natal 59072-970, Brazil; 2 Laboratório de Sistemática e Ecologia de Invertebrados do Solo, Instituto Nacional de Pesquisas da Amazônia—INPA, COBIO/Entomologia, Campus II, Petrópolis, Manaus 69067-375, Brazil; nikolasgc@gmail.com

**Keywords:** chaetotaxy, Entomobryoidea, Heteromurinae, identification keys, review, taxonomy

## Abstract

**Simple Summary:**

Springtails (Collembola) are microarthropods commonly found in terrestrial habitats, especially associated to the soil. *Dicranocentrus* Schött is one of the most representative genera of the family Orchesellidae Börner of springtails, with 69 described species in tropical regions of the world. Here we describe a new Brazilian species of the genus, especially using data on its chaetotaxy, the comparative study of the shape and the arrangement of body chaetae. We also use such data to compare and better understand the morphology of other species of the genus, and to better recognize them using comparative tables and identification keys.

**Abstract:**

*Dicranocentrus* Schött is the most diverse and widespread taxon of Neotropical Orchesellidae. In Brazil, the genus is represented by 11 species found in humid forested areas of Atlantic and Amazon forests domains. Here we describe in detail *Dicranocentrus abestado* sp. nov. from Chapada Diamantina, Caatinga domain, Brazil. The new species belongs to the *marias* group *sensu* Mari-Mutt, due to the absence of most posterior macrochaetae on the dorsal head, and resembles other Neotropical species with 3, 2 and 2 central macrochaetae on abdominal segments I–III. However, the new species is unique especially by its reduced colour pattern combined with its empodial complex morphology. We compare *Dicranocentrus abestado* sp. nov. with 27 other taxa from the New and Old World, including all species with 3 macrochaetae on the first abdominal segment; provide notes and details on the morphology of the compared species plus identification keys to Brazilian and all species of the genus with similar abdominal chaetotaxy. We also discuss the current taxonomical knowledge on Brazilian *Dicranocentrus* and provide notes on its chaetotaxy and Heteromurinae systematics.

## 1. Introduction

Entomobryoidea Womersley, 1934 [1] is the largest superfamily of springtails, with more than 2500 described species [2,3]. The superfamily comprises about 28% of all known Collembola species, and is currently subdivided into three families: Orchesellidae Börner, 1906 [4], Entomobryidae Schäffer, 1896 [5] and Paronellidae Börner, 1906 [4] and *sensu* Zhang et al., 2019 [2]. In Brazil the Entomobryoidea are represented by 159 species, about 36% of all known Brazilian springtails, distributed in all three families and 23 genera [6], of which the family Orchesellidae are represented by two subfamilies, four tribes, five genera and 17 species, summarized in Table 1.

*Dicranocentrus* Schött, 1893 [7] has a Pantropical distribution and is the second largest genus of Orchesellidae, with 69 nominal species, just after *Orchesella* Templeton, 1836 [8], with almost 100 species [3]. *Dicranocentrus* is the most widespread and species-rich genus of Neotropical Orchesellidae [9,10,11]. Its species are epiedaphic, mostly found on ground, over litter, moss, rooting wood and even associated to epiphytes, being rarely found inside termites’ nests [12,13]. The genus’ main diagnostic features are antennae with six segments (antennal segments I and II subdivided), 8 eyes, prelabral chaetae simple (not bifurcate), ungues with a single outer tooth and dental spines, if present, simple (a detailed diagnosis is presented in the results topic) [12,14,15]. Currently, there are 11 species of *Dicranocentrus* known from Brazil, found in humid forested areas of Atlantic and Amazon forests domains (Table 1) [6,13,16,17,18,19,20,21].

Here, we describe in detail a new species of Brazilian *Dicranocentrus* and compare its affinities with 27 other species. We also provide an updated diagnosis to the genus, comparative tables of Brazilian species as well as species with similar abdominal chaetotaxy, discuss the current knowledge on Brazilian *Dicranocentrus*, and provide notes on the chaetotaxy of the genus and Heteromurinae systematics.

## 2. Materials and Methods

Specimens of the new species were preserved in 70% ethanol and sorted with a stereomicroscope Leica S8AP0 (Leica, Wetzlar, Germany). After, they were cleared in Nesbitt’s solution, washed in Arlé’s liquid and mounted on glass slides in Hoyer’s medium, following the procedures described by Arlé and Mendonça [16] and Jordana et al. [33], with few adaptations. Glass slide specimens were studied with Leica DM500 (Leica, Wetzlar, Germany) and DM750 (Leica, Wetzlar, Germany) microscopes, both connected to drawing tubes. The habitus of the new species was photographed with stereomicroscope Nikon SMZ1500 (Nikon, Tokyo, Japan), with a Nikon DS-Ri1 camera (Nikon, Tokyo, Japan) attached, using NIS-Elements AR v.4.51.00 software (Nikon, Tokyo, Japan). Photographs and raw drawings were digitally improved in Corel Photo-Paint X8 (Corel, Ottawa, Canada) and CorelDraw X8 software (Corel, Ottawa, Canada), respectively.

The chaetotaxy terminology used in this study follows mainly Fjellberg [34] to labial palp papillae; Gisin [35] to labial chaetotaxy with additions of Zhang and Pan [36]; Cipola et al. [37] to labral chaetotaxy; Mari-Mutt [12] to dorsal head chaetotaxy with additions of Soto-Adames [38] and Zhang et al. [15]; Szeptycki [39] and Zhang and Deharveng [26] to S-chaetotaxy; an Szeptycki [40] to dorsal chaetotaxy, with additions and modifications provided by Soto-Adames [38], Cipola et al. [14] and Zhang et al. [2,41].

The abbreviations used in the descriptions are: Abd.—abdominal segment(s); Ant.—antennal segment(s); mac—macrochaeta(e); mes—mesochaeta(e); mic—microchaeta(e); **ms**—S-microchaeta(e); sens—ordinary S-chaeta(e); and Th.—thoracic segment(s). Antennal segments I and II subdivisions are: "a" to proximal subarticle, "b" to distal one. Depository abbreviation: CC/UFRN—Collembola Collection of the Biosciences Center of Federal University of Rio Grande do Norte, Brazil; INPA—Invertebrate Collection of the National Institute of Amazonian Research, Manaus, Brazil.

The symbols used in the drawings to represent the dorsal chaetotaxy schemes are: large blank circles to mac; large black circles to mes; small black circles to mic; blank circles with small black circle inside to mac or mic; black chaetae-like drawings to sens or **ms**; black circles crossed by a line for pseudopores; bothriotricha-like drawings to bothriotricha; a dash above or under any symbol to chaetae present or absent. Taxonomic description and comparisons are based on half body. Chaetae labels (including rows) and labial papillae are marked in bold in the text.

## 3. Results

### 3.1. Taxonomic Summary and Genus Diagnosis

Order Entomobryomorpha Börner, 1913 [42]

Superfamily Entomobryoidea Womersley, 1934 [1]

Orchesellidae Börner, 1906 [4] *sensu* Zhang et al. 2019 [2]

Family Heteromurinae Absolon and Ksenemann, 1942 [22] *sensu* Zhang and Deharveng, 2015 [26]

Tribe Heteromurini Absolon and Ksenemann, 1942 [22] *sensu* Zhang et al. 2020 [15]

Genus *Dicranocentrus* Schött, 1893 [7]

Diagnosis: Specimens mostly pigmented, colour patterns variable, eyepatches black. Antennae six-segmented, Ant. I and II basally subdivided, proximal subarticles smaller than the distal ones, Ant. III usually longer than Ant. IV, rarely subequal or smaller; apical bulb of Ant. IV absent; Ant. III–IV annulated, devoid of scales. Postantennal organ absent. Eight eyes. Prelabral chaetae smooth and simple (not bifurcate). Labial chaetae **e** smooth; post-labial quadrangle (anterior chaetae surrounding the cephalic groove) chaetae smooth or rough, never ciliate. Th. II not projected over head. Abd. V with 4 or 7 sens (atypical). Ungues with a single outer tooth. Simple dental spines present or absent. Mucronal spine present. Data adapted and modified of Mari-Mutt [12], Cipola et al. [14], Zhang et al. [15] and Xisto and Mendonça [43].

Type species: *Dicranocentrus gracilis* Schött, 1893 [7]

Remarks: The presence of rounded and/or truncate coarsely ciliate scales on body and appendages, sens and **ms** formulae of dorsal Th. II to Abd. III = 2,2| 1,3,3 and 1,0| 1,0,1, respectively, short Abd. IV (less than two times the length of the Abd. III at the midline) and bidentate mucro were suppressed from the genus diagnosis since they are suprageneric diagnostic features. The updated diagnosis changes the number of sensilla on Abd. V compared to previous literature, as in [2,14,15], due to the description of the new species. More details on *Dicranocentrus* overall morphology are presented in [12,14,15,43,44,45].

### 3.2. Dicranocentrus abestado sp. nov. Siqueira, Bellini, and Cipola

**Figure 1:**
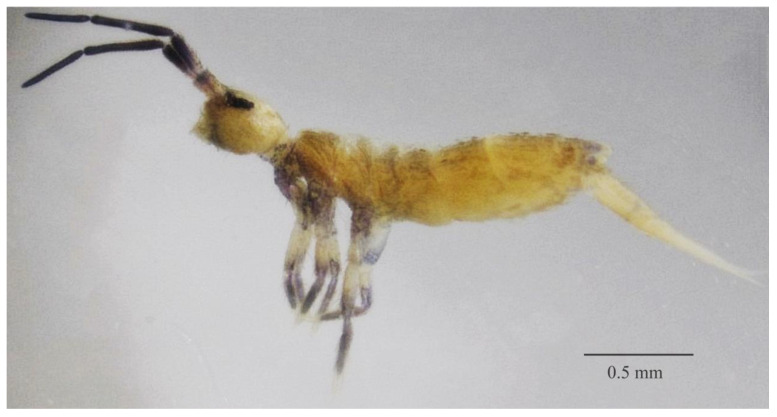
*Dicranocentrus abestado* sp. nov. habitus, specimen fixed in ethanol (lateral view).

**Figure 2:**
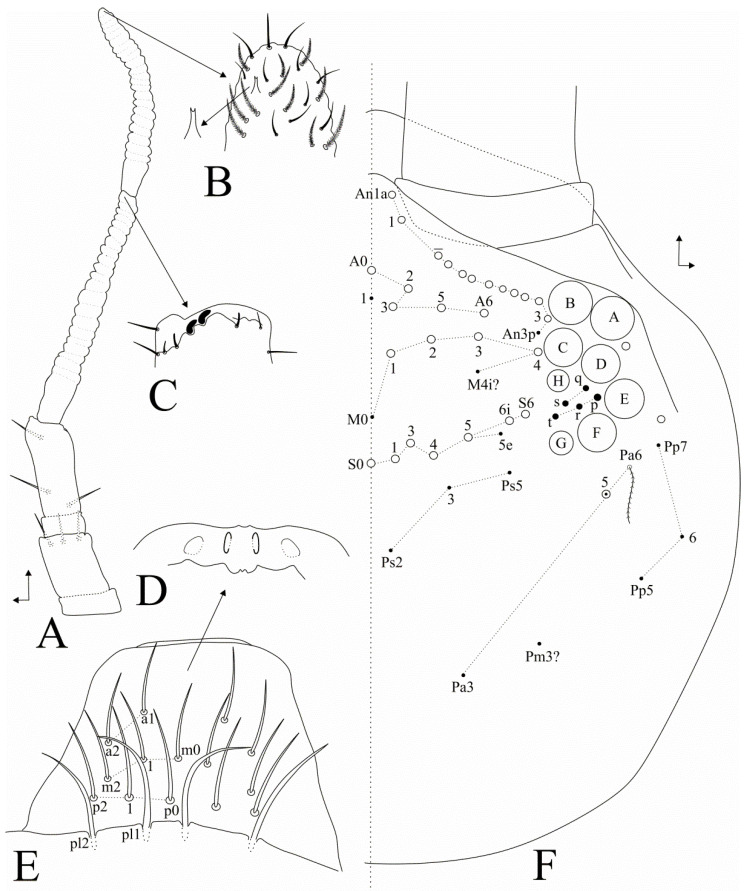
*Dicranocentrus abestado* sp. nov. head: (**A**) left antenna (dorsal view); (**B**) apical Ant. IV (ventro-lateral view), arrow indicates bifurcate pin projection; (**C**) apical organ of Ant. III (ventro-lateral view); (**D**) labral papillae; (**E**) labral and pre-labral chaetotaxy; (**F**) dorsal head chaetotaxy and eyes (right side).

**Figure 3:**
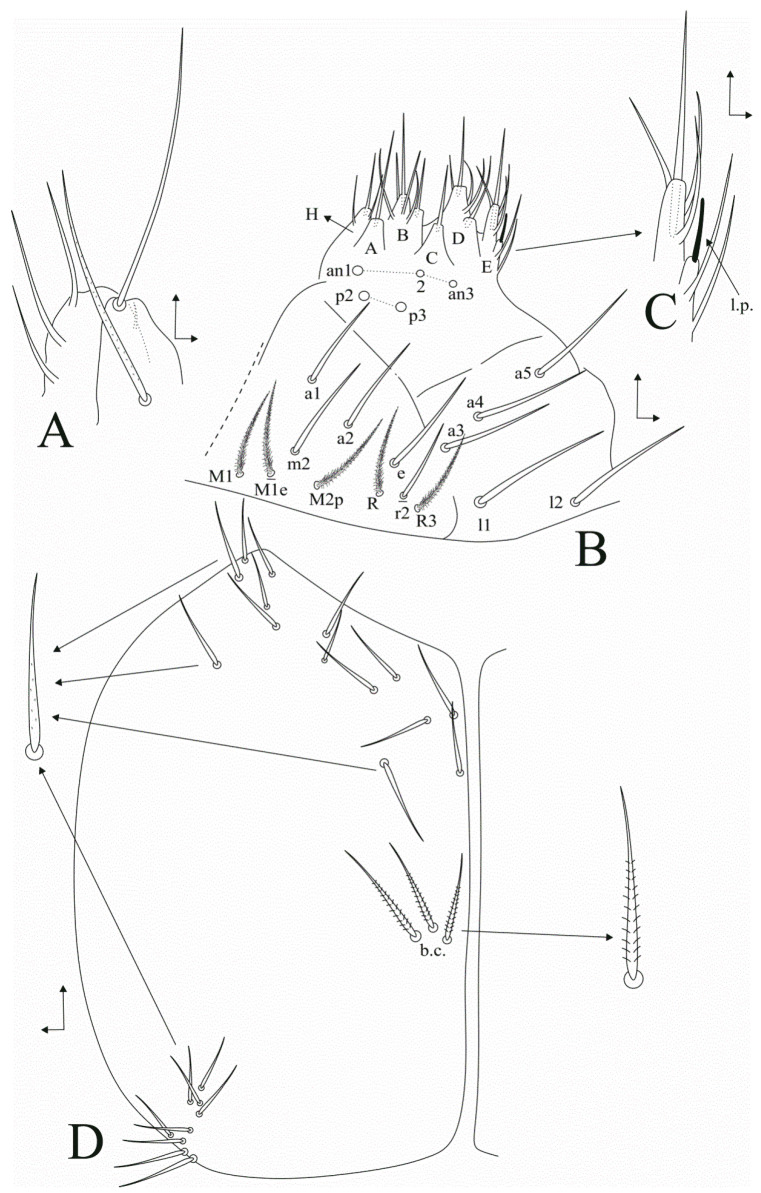
*Dicranocentrus abestado* sp. nov. ventral head: (**A**) maxillary outer lobe and sublobal plate (right side); (**B**) labium (right side); (**C**) labial papilla **E** (right side), arrow indicates lateral process (**l.p.**); (**D**) post-labial chaetotaxy (left side), arrows indicate morphology of chaetae, (**b.c.**) = basal chaetae.

**Figure 4:**
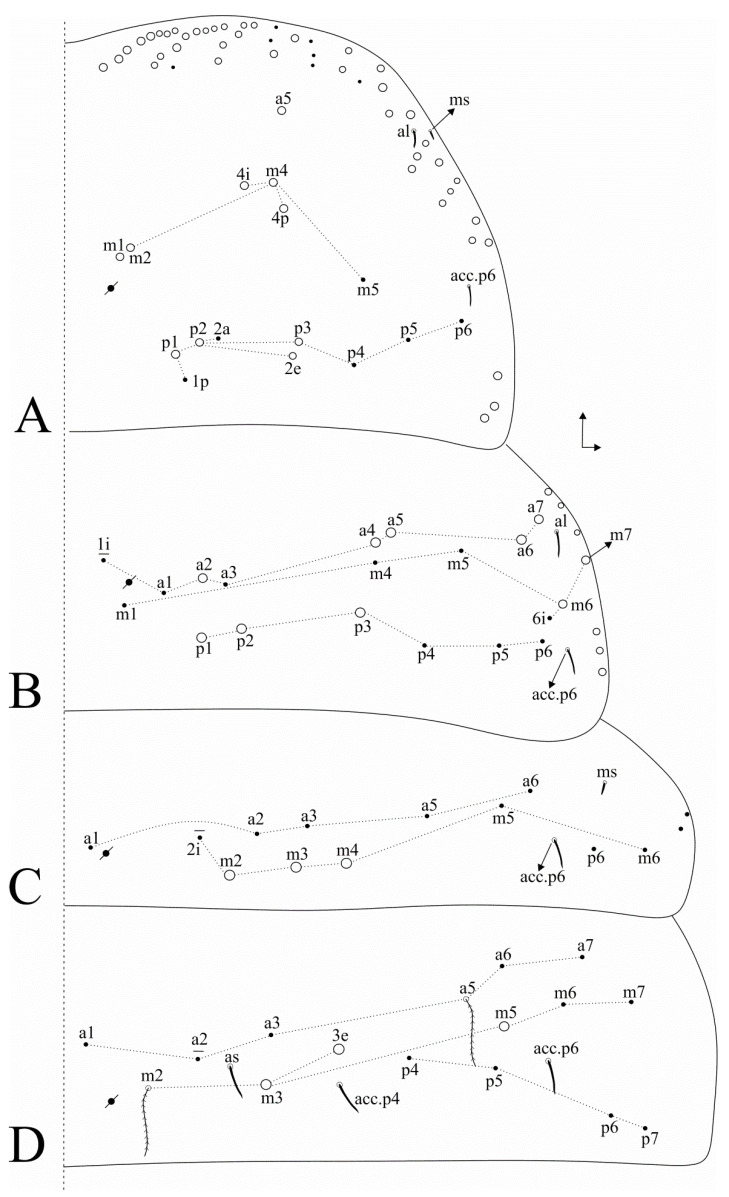
*Dicranocentrus abestado* sp. nov. trunk dorsal chaetotaxy (right side): (**A**) Th. II; (**B**) Th. III; (**C**) Abd. I; (**D**) Abd. II.

**Figure 5:**
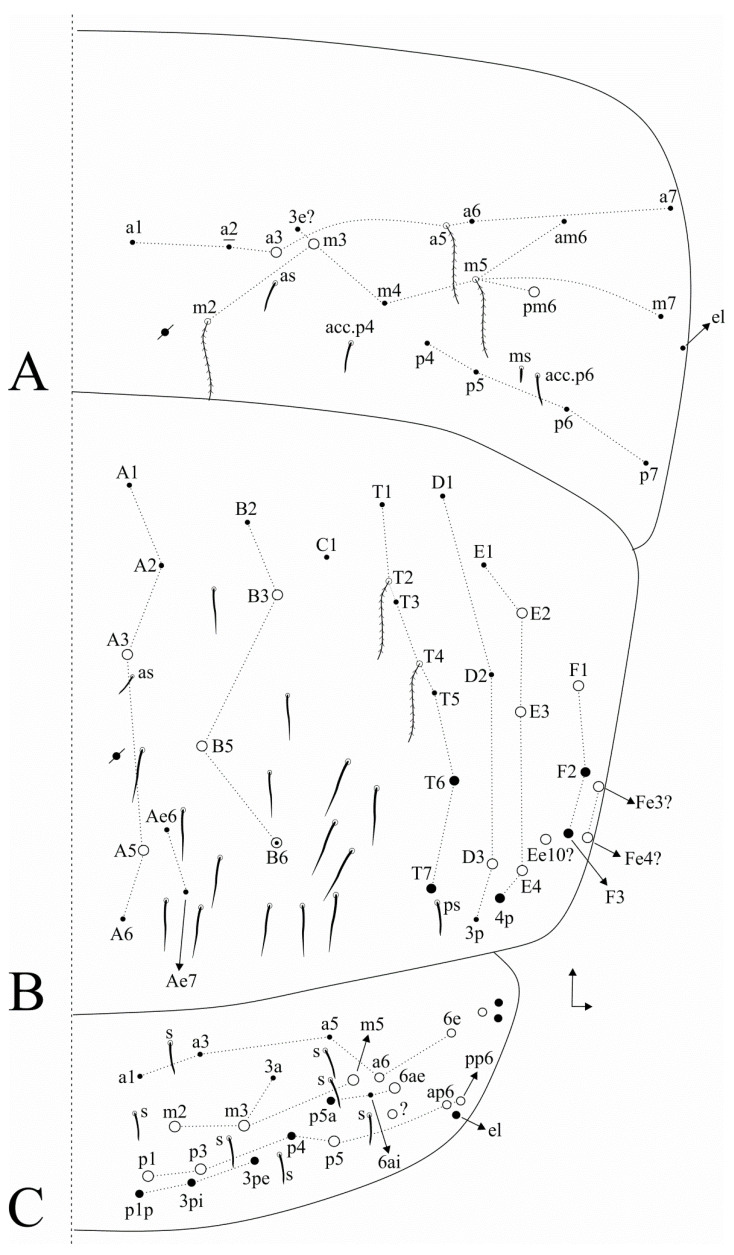
*Dicranocentrus abestado* sp. nov. trunk dorsal chaetotaxy (right side): (**A**) Abd. III; (**B**) Abd. IV; (**C**) Abd. V.

**Figure 6:**
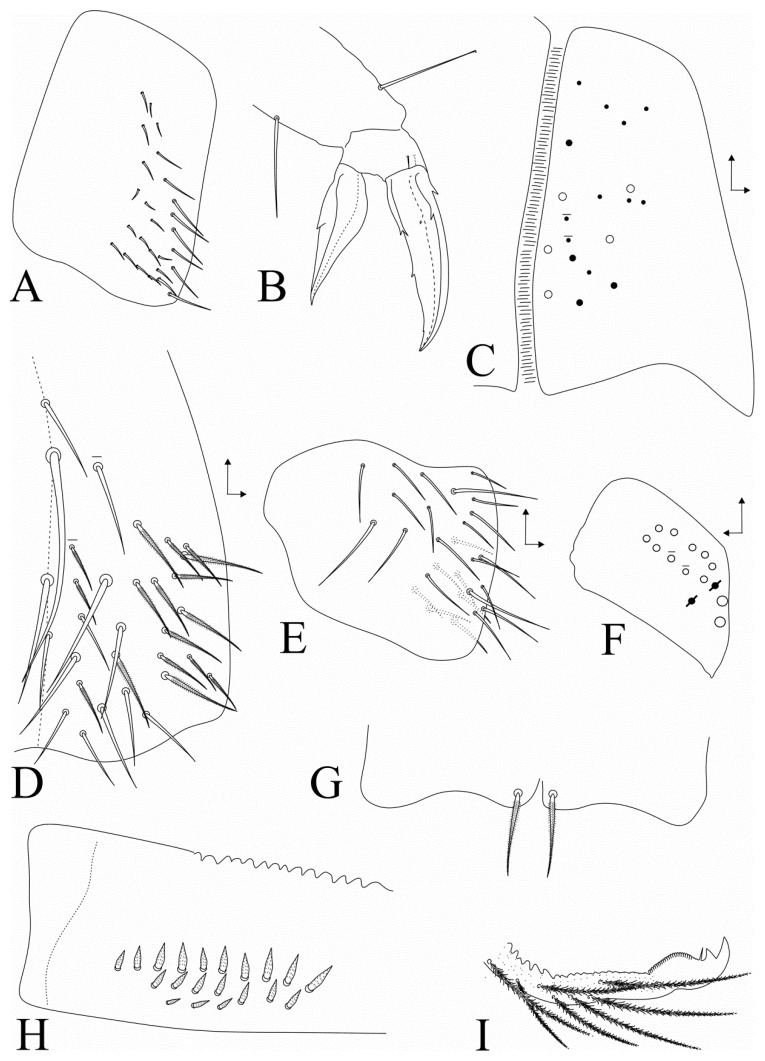
*Dicranocentrus abestado* sp. nov. trunk appendages: (**A**) trochanteral organ; (**B**) empodial complex of leg. III (anterior view); (**C**) ventral tube anterior face (right side); (**D**) ventral tube posterior face (right side); (**E**) ventral tube lateral flap (right side); (**F**) manubrial plate (left side); (**G**) manubrium ventro-distal chaetae; (**H**) spines on basal dens (internal view of right side); (**I**) distal dens and mucro.

Figure 1, Figure 2, Figure 3, Figure 4, Figure 5 and Figure 6, Table 2 and Table 3

Type material: Holotype: Female in slide (CC/UFRN): Brazil, Bahia State, Chapada Diamantina National Park, Lençóis Municipality (12°33′47″ S, 41°23′28″ W), Caatinga phytogeographic domain, 07.xi.2013, entomological aspirator, B.C. Bellini coll. Five paratypes in slides (CC/UFRN): two males, one female and two juveniles, plus one female (INPA-CLL 0000114), same data as holotype.

Diagnosis: Bluish pigments on Ant. II–IV, Th. II–III laterally to coxae, Abd VI, distal femurs and tibiotarsi. Ant. III longer than Ant. IV, Ant. IIb and Ib with three ventral smooth acuminate chaetae each. Labral papillae rounded. Dorsal head **An**, **A**, **M**, **S** and **Ps** series with 11–12, 5, 4, 7 and 0 mac, respectively; 0–1 posterior mac (**Pa5** present or absent), five interocular chaetae. Maxillary outer lobe basal chaeta acuminate, labial papilla **E** lateral process (**l.p.**) finger-shaped, not reaching the papilla apex. Labial chaetae **m2**, **r2** (if present) and **l2** smooth, **M1**–**1e** (if present), **M2p**, **R** and **R3** ciliate, labium unscaled. Three weakly ciliate post-labial basal chaetae (**b.c.**). Th. II with five **m** and four **p** main mac, **p5** as mic; Th. III with 3, 0, 3 central mac on rows **a**, **m** and **p**, respectively; Abd. I with three central mac; Abd. III with two central and one lateral mac; Abd. IV with 4–5 central mac, **B6** as mic or mac; Abd. V with seven sens. Trochanteral organ with 22–27 chaetae. Tibiotarsi I–II with 3, tibiotarsus III with five distal smooth large chaetae on inner side, respectively. Tenent hairs weakly capitate or acuminate; ungues with one tiny apical tooth; unguiculi lanceolate, leaf shaped, antero-internal lamella wide, postero-external with one tooth. Ventral tube anterior face distally with one mac and two mes, posterior face with four unpaired plus 9–10 paired smooth chaetae, lateral flap with about 27 smooth chaetae. Tenaculum corpus with one rough chaeta. Manubrium with three and dens proximally with one dorsal smooth acuminate chaetae, manubrial plate with 10–12 ciliate chaetae plus two pseudopores, manubrium ventro-apical region with one ciliate chaeta. Dens with 16–20 spines distributed in 2–3 rows in proximo-internal region.

Description: Habitus typical of the genus (Figure 1). Body length (head + trunk) of holotype = 1.57 mm; range of type series length (adult specimens) = 1.48–1.72 mm; average body length of adult females = 1.59 mm; average body length of adult males = 1.51 mm; average body length of adults = 1.56 mm. Specimens fixed in ethanol with yellowish background, Ant. I distally and Ant. II–IV dark blue, frontal head (clypeal and anterior post-labial regions), lateral Th. I–III, dorsal Abd. VI, coxae (including epi and subcoxae), distal half of femurs and proximal 3/4 of tibiotarsi light blue (Figure 1). Coarsely ciliate scales present on Ant. I, Ant. IIa, head (dorsally and ventrally), dorsal trunk, legs (all segments), ventral tube anterior and posterior faces, ventral manubrium and ventral dens. Dorsal head and trunk mac, mes and mic ciliate.

Head (Figure 2 and Figure 3). Antennae shorter than trunk (Figure 1 and Figure 2A), ratio antennae: Body = 1:1.59 (holotype), average 1:1.81 (adult specimens); Ant. III longer than Ant. IV, antennal ratio Ant. Ia–IV of holotype = 1:2.2:1:3.7:9.5:7.5; antennal ratio of type series adults = 1:2.2–5:1–2:3.7–7:8.7–9.5:6.7–17.3. Ant. IV subapical organite not seen, apparently missing; Ant. IV with a ventral subapical bifurcated pin projection, plus sens and ciliate chaetae (Figure 2B). Ant. III sense organ with 2 enlarged sensory rods, three guard sensilla plus at least four surrounding acuminate sens (Figure 2C). Ant. IIb and Ib with three ventral smooth acuminate chaetae each (Figure 2A). Labral papillae rounded, internal marked and more oval, external more discrete (Figure 2D); Labral formula with four (**a1–2**), 5 (**m0**–**2**), 5 (**p0**–**2**) smooth chaetae, **p0**–**2** slightly longer than others, **a** chaetae slender, prelabral chaetae larger than the labral ones (Figure 2E). Eyepatches largest lenses A and B, C–F subequal, G–H smaller than the others, with five interocular mes (Figure 2F). Head dorsal chaetotaxy with antennal (**An**) row with 11–12 mac, anterior (**A**) row with five (**A0**, **A2**–**3**, **A5**–**6**), medial (**M**) row with four (**M1**–**4**); sutural (**S**) row with seven (**S0**–**1**, **S3**–**6**), and post-occipital anterior (**Pa**) row with 0–1 (**Pa5** present or absent) mac (Figure 2F). Maxillary outer lobe basal chaeta acuminate, slender and rough, slightly smaller than the apical smooth one, ratio basal chaeta:apical chaeta of holotype = 1:1.1; sublobal plate with four chaeta-like appendages, lateral one reduced, interno-basal one smaller than the interno-apical ones (Figure 3A). Labium with five main papillae (**A**–**E**), with 0, 5, 0, 4, 5 guard chaetae, respectively, papilla **E** lateral process (**l.p.**) finger-shaped, not reaching the papilla apex (Figure 3B,C); five proximal chaetae, **an2**–**3** slightly smaller than others (Figure 3B); labial basomedial (submentum or labial triangle) and basolateral (mentum) fields with chaetae **a1**–**5**, **m2**, **e**, **r2** (present or absent), **l1**–**2** smooth; **M1**–**1e** (present or absent), **M2p**, **R** and **R3** ciliate; labium unscaled (Figure 3B). Post-labial chaetotaxy with 14 rough, almost smooth, anterior chaetae, post-labial quadrangle with two chaetae, three basal chaetae slightly ciliate, posterior head with about nine grouped rough chaetae (Figure 3D).

Trunk dorsal chaetotaxy (Figure 4 and Figure 5). Th. II, excluding anterior collar, with one anterior (**a5**), five medial (**m1**–**2**, **m4**–**4p**) and four posterior (**p1**–**3**) mac, **p5** as mic (Figure 4A). Th. III with five anterior (**a2**, **a4**–**7**), two medial (**m6**–**7**) and three posterior (**p1**–**3**) mac (Figure 4B). Abd. I with three medial (**m2**–**4**) mac (Figure 4C). Abd. II with three medial (**m3**–**3e**, **m5**) mac (Figure 4D). Abd. III with one anterior (**a3**) and two medial (**m3**, **pm6**) mac (Figure 5A). Abd. IV with 4–5 central (**A3**, **A5**, **B3**, **B5**–**6**, **B6** as mac or mic) and eight lateral (**D3**, **E2**–**4**, **Ee10?**, **F1**, **Fe3?**–**Fe4?**) mac, **as** sens just under **A3**, at least 14 central long sens (possibly many more) (Figure 5B). Ratio Abd. III:IV of holotype 1:1.7, ratio Abd. III:IV of type series adults 1: 1.5–1.7. Abd. V macrochaetotaxy with two anterior (**a6**–**6e**), three medial (**m2**–**3**, **m5**), one postero-anterior (**p6ae**), five posterior (**p1**, **p3**, **p5**, **ap6**, **pp6**) plus one mac without clear homology (**?**), four internal and three lateral sens (Figure 5C). More details on idio and S-chaetotaxy are represented in Figure 4 and Figure 5.

Legs (Figure 6A,B): Trochanteral organ with 22–27 spine-like smooth chaetae, 22 on holotype (Figure 6A). Distal halves of tibiotarsi I–II with three and tibiotarsus III with five smooth chaetae on inner side, respectively. Tenent hairs discretely capitate or acuminate, two small pretarsal chaetae present, tibiotarsus III distal smooth chaeta subequal in length to tenant hair (Figure 6B); empodial complex III ratio of smooth chaeta: tenent hair: unguiculus: unguis of holotype as 1:1:1.5:2.2. Ungues with an outer pair of lateral undeveloped teeth on proximal 1/4, inner side with four teeth: two paired basal teeth on proximal 1/3, one unpaired median tooth on proximal 3/5 with the same size of the basal teeth, and a minute apical tooth on distal 1/8 easily overlooked due to size and position; unguiculi lanceolate, leaf shaped, antero-internal lamella wide, all lamellae smooth except postero-external lamella with a large tooth on proximal 1/2 (Figure 6B).

Abdominal appendages (Figure 6C–I): Ventral tube anterior face with about 17–19 ciliate chaetae, five mac and 12–14 slender chaetae with different sizes, distal region with one mac and two mes (Figure 6C); posterior face with four unpaired (one very large) and 9–10 paired smooth chaetae with different sizes, plus 3–4 central ciliate chaetae (lateral side with many more ciliate chaetae, partially represented in Figure 6D); lateral flap with about 27 smooth chaetae, three clearly longer than the others (Figure 6E). Tenaculum corpus with a single rough chaeta, each ramus with four teeth. Dorsal manubrium with three smooth acuminate chaetae, one at the base, one median and one subapical; manubrial plate with 10–12 ciliate chaetae (2 inner as mac) plus two pseudopores (Figure 6F); manubrium ventro-apical region with one ciliate chaeta (Figure 6G). Dorsal dens with one smooth acuminate chaeta; dens with 16–20 rough spines (18–20 in holotype) in 2–3 rows in proximo-internal region (Figure 6H). Mucro apical tooth larger than the basal one, mucronal spine reaching the apex of the basal tooth (Figure 6I).

Etymology: “abestado” or “abestada” is a regional expression used in Northeastern Brazil which means “fool”. The expression is commonly used between friends.

Habitat: The new species was found in Chapada Diamantina National Park, in the southern region of Caatinga phytogeographic domain, from moist rocky and sandy soil samples surrounded by forested areas. The climate of the area is “Aw” according to the Köppen–Geiger system—an equatorial climate with dry desert-like summer [46]. The specimens were collected at the beginning of the raining season.

Remarks: *Dicranocentrus abestado* sp. nov. belongs to the *marias* group of species as its dorsal head chaetotaxy is devoid of **A1**, **S2** and all posterior mac except **Pa5** and rarely **Pp5** [12,47]. The new species is most similar to other Neotropical taxa from *marias* group, especially *D. antillensis* Mari-Mutt, 1979 [12], *D. icelosmarias* Soto-Adames and Anderson, 2017 [48], *D. marias* Wray, 1953 [49] and *D. paramoensis* Mari-Mutt, 1983 [47] since they also share seven mac on dorsal head **S** row (**S0**–**1**, **S3**–**6**), labial papilla **E** lateral process (**l.p.**) short, not reaching the apex of the papilla, labial basomedian field (labial triangle) with **M1** chaeta ciliate and **m2** and **e** smooth, Th. II lacking **p5** mac and Abd. I–III with 3, 2 and 2 central mac, respectively. However, the new species can be readily separated from all others due to its colour pattern, with bluish pigments on thorax laterally to coxae, Abd. VI, distal femurs distally and all tibiotarsi (otherwise in the other species) and by its wide leaf-shaped unguiculus (normal in the other species), with one posterior tooth (absent in *D. icelosmarias*) (Table 2). The new species also lacks **Pp5** mac on dorsal head (present in *D. paramoensis*), labial basomedian field unscaled with **R** chaeta ciliate (with scales in *D. antillensis*, **R** chaeta smooth at least in *D. icelosmarias* and *D. marias*), 3 basal post-labial basal chaetae around the cephalic groove (2 in *D. paramoensis*), Th. II with 3 **m4** and 4 **p** mac (2 and 5, respectively, in *D. antillensis* and *D. paramoensis*), Th. III with 3, 0, 3 mac on **a**, **m** and **p** rows, respectively (4, 1, 3 in *D. antillensis* and 2, 1, 3 in *D. paramoensis*), unguis minute apical tooth (absent in *D. antillensis*, *D. icelosmarias* and *D. marias*) and dorsal manubrium with 3 smooth acuminate chaetae (absent in *D. antillensis*, 4 in *D. icelosmarias* and *D. paramoensis*). A detailed comparison of the main diagnostic features among such species is presented in Table 2.

Regarding the Brazilian species of *Dicranocentrus*, *D. abestado* sp. nov. is most similar to *D. amazonicus*, also from the *marias* group (Table 3). These species share similar colour patterns and dorsal head macrochaetotaxy, maxillary outer lobe basal chaeta acuminate, basomedian labial field unscaled with **M1** chaeta ciliate, Th. II **p** row with four mac and Th. III **a**, **m**, **p** rows with 3, 0, 3 mac, respectively. However, the new species differs from *D. amazonicus* especially by labial chaeta **R** ciliate (smooth in *D. amazonicus*), Abd. II with 2 central mac (1 in *D. amazonicus*), Abd. III with 1 lateral mac (2 in *D. amazonicus*), Abd. IV with 4–5 central mac (3 in *D. amazonicus*), unguiculus wide with one posterior tooth (normal and toothless in *D. amazonicus*) and unguis with a minute apical tooth (absent in *D. amazonicus*). *Dicranocentrus pikachu* is the second previously known species of the *marias* group from Brazil; however, it is remarkably different from *D. abestado* sp. nov., especially as it has 17 dorsal head **An** mac (11–12 in the new species), maxillary outer lobe basal chaeta blunt (acuminate in the new species), Th. II with 5–6 **p** chaetae (4 in the new species), Th. III **a**, **m**, **p** rows with 5, 1, 3 mac, respectively (3, 0, 3 in the new species), Abd. I with 5 central mac (vs. 3), Abd. III with 2 lateral mac (vs. 1), unguis lacking the apical tooth (vs. present) and about 50 dental spines (vs. 16–20).

Among the Brazilian species, *D. abestado* sp. nov. is the only one with 3 mac on Abd. I (Table 3). *Dicranocentrus heloisae* typically has 5 mac on Abd. I [18]; however, it was reported in specimens also with 3 [21]. Here, we disregard such observations, since it is possible that specimens of *D. heloisae* with 3 mac on Abd. I represent another species, as the macrochaetatoxy of Abd. I is quite a stable feature in *Dicranocentrus*; or such specimens may represent juveniles. Even so, *D. abestado* sp. nov. also differs by head with 0–1 posterior mac (*marias* group), while in *D. heloisae* there are 2 (*gracilis* group). In addition, *D. heloisae* has a very distinct colour pattern, plus large number of chaetae in trochanteral organ and dental spines, compared to the new species. Further comparisons between both species and among other Brazilian taxa are presented in Table 3 and in the identification key in the discussion.

**Figure 7:**
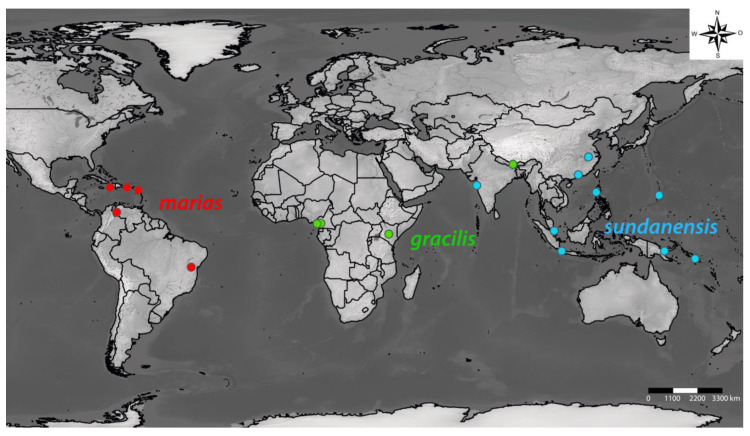
Type localities of all 18 *Dicranocentrus* species with 3 mac on Abd. I, distributed in the *marias* group (red dots, Neotropical region) and in the *gracilis* and *sundanensis* groups (green and blue dots, respectively, Paleotropical region). Further data on such species are presented in Table 2, Table 4 and Table 5.

*Dicranocentrus abestado* sp. nov. also resembles species from the Old World from *sundanensis* and *gracilis* groups with 3 mac on Abd. I, especially *D. gemellus* Mari-Mutt 1985 [50], *D. indicus* Bonet, 1930 [51], *D. inermodentes* (Uchida, 1944) [52], *D. luzonensis* Mari-Mutt, 1985 [50], *D. nepalensis* Mari-Mutt and Bhattacharjee, 1980 [53], *D. solomonensis* Mari-Mutt, 1979 [12] and *D. wangi* Ma and Chen, 2007 [54] also by Abd. II–III central macrochaetotaxy with 2 and 2 mac, respectively. However, the Old World species have a more complex macrochaetotaxy on posterior head, which dismiss them from the *marias* group (head chaetotaxy unknown to *D. indicus*), as well as: 6–10 mac on **p** row of Th. II (4 in the new species), 9–12 mac on central Th. III (6 in the new species) and dental spines absent (except in *D. luzonensis* and *D. wangi*—also present in the new species). A detailed comparison among such species is presented in Table 4. Compared to species from the Old World from the *sundanensis* and *gracilis* groups with Abd I–II with 3 and 2 mac, respectively, but with 1 central mac on Abd. III instead (*D. assimilis* Schött, 1927 [55], *D. inermis* Schött, 1927 [55], *D. javanus* Yoshii and Suhardjono, 1989 [56], *D. liuae* Xu and Zhang, 2014 [57], *D. meruensis* Wahlgren, 1908 [58] and *D. simplex* Yosii, 1959 [59]), the new species differs beyond dorsal head and central Abd. III macrochaetotaxy, by having Th. II with 3 **m4** (2 in the others except in *D. assimilis*) and 4 **p** mac (6–9 in the others), Th. III with 6 central mac (7–11 in the others), unguis with a minute apical tooth (absent in the others) and 16–20 dental spines (present only in *D. meruensis*—about 40–46 spines; in *D. simplex*—5–8 spines). Such species are compared in detail in Table 5. The type localities of all *Dicranocentrus* species with 3 mac on Abd. I are shown in Figure 7.

## 4. Discussion

### 4.1. Brazilian Species of Dicranocentrus

The knowledge on Brazilian *Dicranocentrus* further increased during the past decade but is still limited at some level. With the description of *D. abestado* sp. nov. there are now 12 species of the genus recorded from Brazil (Table 1). Most of these species are only known from their type locality, with the exception of *D. albicephalus* (from Rio de Janeiro and Espírito Santo states), *D. heloisae* (from Rio de Janeiro, Espírito Santo, São Paulo, Minas Gerais and Bahia states) and *D. silvestrii* (Rio de Janeiro, Bahia and possibly Santa Catarina states) [4,19,20,21,61,62,63]. Of such species only *D. silvestrii* was recorded in other countries and it is considered widespread in South America, known from Argentina, Bolivia, Brazil, Chile, Peru and Venezuela [9], although its records are doubtful (see comments on this species, below). There are also few records of unidentified morphospecies from Brazil, as in [62,64], which combined to the nominal species records suggest that *Dicranoncentrus* is widely distributed at least in the Atlantic and Amazon Forest domains, as well as humid forested areas within the Caatinga domain.

Concerning the morphology of Brazilian species, *D. albicephalus*, *D. cuprum*, *D. heloisae*, *D. magnus*, *D. marimutti*, *D. melinus* and *D. pikachu* were very well studied and described/redescribed by Xisto and Mendonça [18,20,21]. Very few diagnostic features are unknown to these species, such as the number of smooth chaetae on furca and trunk sens formula, for example. Unfortunately, the type/analysed material of such species was lost in the fire in the National Museum of Rio de Janeiro at the end of 2018. The description of *Dicranocentrus amazonicus* is succinct as to most species of the genus, and some diagnostic features are unknown or unclear to it, such as the presence and distribution of smooth chaetae on antennae, tibiotarsi and furca, trunk sens formula, antennal and ventral head chaetotaxy (except labial and maxillary outer lobe chaetotaxy), number of tenaculum chaetae, as well as other features. The labral papillae shape and position and absence of scales on ventral tube are also unlikely characters and should be better investigated in this species [17]. Nevertheless, there are enough data to separate this species from other Brazilian and Neotropical taxa (Table 2 and Table 3). *Dicranocentrus bicolor*, *D. termitophilus* and *D. silvestrii*, however, are poorly described and their names cannot be used with confidence for now, as already stated by Mari-Mutt [12] and Xisto and Mendonça [21]. Because of this we considered the three species as *species inquirendae*.

*Dicranocentrus bicolor* was collected in the Blumenau municipality, Santa Catarina state, inside nests of *Eutermes arenarius* termites [13]. Even though the type material of this species is in poor condition [12], there are sufficient data on the type locality and morphology, such as colour pattern, empodial complex and proximal dens—See Table 3 and Handschin ([13], pp. 23–24)—which allow for the collection of fresh samples, recognition of the species and its redescription. *Dicranocentrus termitophilus*-type material is also in poor condition [12], but the two type specimens were collected from Minas Gerais state—wrongly spelled “Minas Gueras” by Handschin [13]—inside nests of *Cornitermes similis* termites. Minas Gerais is the fourth largest state in Brazil with an area of about 586,500 square kilometres and encompass three very distinct biomes: Atlantic Forest (a tropical rainforest); Caatinga (a semi-arid landscape covered by a mosaic of very different phytophysiognomies) and Cerrado (a savanna-like domain). However, the few details on *D. termitophilus* morphology—see Table 3 and Handschin ([13], pp. 25–26)—the overall affinity of Brazilian *Dicranocentrus* species for humid forested areas (Table 1) and the biological association, even if accidental, with *Cornitermes similis* may provide enough data for the collection of new samples and the redescription of *D. termitophilus*. The very brief and generic description of *D. silvestrii* by Absolon ([24], pp. 105–106) makes it impossible to clearly separate this species from most taxa, and so we excluded it from Table 3. For instance, the body colour is described as yellowish, but also as dark in one species variation, without further details. Absolon’s description also reports a capitate tenent hair (seen in several species of the genus and in a few polymorphic ones, as shown in Table 2, Table 3, Table 4 and Table 5) and unguiculus with a medial tooth (also seen in several species, as shown in Table 2, Table 3, Table 4 and Table 5). However, the most obscure data for this species is its type locality, which is listed only as “in South America” ([24], p. 106). Since its type material is apparently lost [12], the real type locality is unknown and none of the posterior descriptions consulted the type material [65,66], the name *D. silvestrii* is highly dubious and should not be used in any case. One last detail about this species is the record of Börner [4] to *D. silvestrii* on orchids from “São Francisco”, Brazil. This locality is as dubious as the species name, since “São Francisco” could represent several different localities far apart in Brazil. Further details on this issue are discussed in ([67], pp. 161).

**Table 4:** Comparison among *Dicranocentrus* species from the Old Word with Abd. I–III with 3, 2 and 2 central mac, respectively.

	Species [References]	*gemellus*	*indicus*	*inermodentes*	*luzonensis*	*nepalensis*	*solomonensis*	*wangi*
		[50]	[12,51,54,60]	[12,44,52]	[50]	[53]	[12,44]	[54]
	Type Locality	Finschhafen,	Bandra,	Saipan,	Baguio,	Chainpur,	Guadalcanal,	Guangzhou,
		Papua New Guinea	India	Northern Mariana Is.	Philippine Is.	Nepal	Solomon Is.	China
Characteristics	Species Group	*sundanensis*	*sundanensis*?	*sundanensis*	*sundanensis*	*gracilis*	*sundanensis*	*sundanensis*
Body bluish/violetish		entire body or just	Ant., lateral Th. II	Ant., anterior head	Ant. and legs	Ant. and	entire body, except	Ant., distal
pigmentation on		antennae and legs	and posterior trunk segments	and legs	(except femurs)	anterior head	dorsal head and	femurs and
			segments				furcula	tibiotarsi
Interocular chaetae		4–5	?	?	3	?	?	?
Dorsal head mac	**S2**	+	?	+	+	‒	+	+
	**Pa1**	+	?	+	‒	+	+	‒
	**Pa2**	+	?	+	+	+	+	+
	**Pp3**	+	?	+	‒	+	+	+
	**Pp5**	+	?	+	+	‒	+	+
Labral papillae	inner	hooked	?	conical	conical	conical	conical	conical
	outer	hooked	?	conical	rounded	conical	conical	conical
Labial basomedian field	**M1**	S	?	C	C	C	S	C
	**M2**	S	?	C	S	C	S	S
	**R**	S	?	S/C	S	C	S	S
	extra chaetae	2–3 S	?	0–1 S, 3–4 C	0–2 S, 1–2 C	2 C	4–6 S	0–1 S, 2 C
Th. II mac	**p1–3** group	8	8	7	8	6	8	7–9
	**p5** mac	‒	‒	+	‒	‒	‒	+
Th. III central mac	**a, m, p** rows	5,1,3	5,1,3	5,1,3	5,1,3	5,1,3	5,1,5	5,2,5
Abd. IV central mac		4–5	?	3	3	4	4–5	4–5
Trochanteral organ chaetae		30–36	29	?	24–37	?	?	25–41
Tibiotarsus III	inner	+(several)	‒	‒	15–20	‒	+?	0–2
smooth chaetae	outer	+(several)	‒	‒	1–2	‒	+?	5–11
Tenent hair		capitate/acuminate	capitate	capitate	acuminate	capitate	acuminate	acuminate
Unguis teeth	ratio	b.t. = m.t.>a.t.	b.t. > m.t.	b.t. = m.t.	b.t. > m.t. = a.t.	b.t. > m.t. = a.t.	b.t. = m.t.	b.t. > m.t.
	**a.t.**	+/‒	–	‒	+/‒	+	‒	‒
Unguiculus	**pe**	0–1 tooth	smooth	smooth	1 tooth	1 tooth	1 tooth	0–1 tooth
Tenaculum chaetae chaetae		3–4	1	?	5–7	?	?	4–10
Manubrium dorsal	number	3–4	‒	‒	5–6	‒	5	23–25
smooth chaeta	distribution	2 rows	‒	‒	2 rows	‒	2 rows	3 rows
Dens (basal) smooth chaeta		1	?	‒	1–3	‒	1	1–4
Dens (dorsal) blunt mac		‒	+	‒	‒	‒	‒	‒
Dental spines	number	‒	‒	‒	14–20	‒	‒	8–13
	distribution	‒	‒	‒	1 row	‒	‒	1 row

Legends: [] references; (+) present; (‒) absent; (/) or; (?) unknown/unclear; (S) smooth; (C) ciliate; ungual teeth = (b.t.) basal paired teeth, (m.t.) medial unpaired tooth, (a.p.) apical unpaired tooth; unguiculus lamellae = (pe) postero-external lamella.

**Table 5:** Comparison among *Dicranocentrus* species from the Old Word with Abd I–III with 3, 2 and 1 central mac, respectively.

	Species [references]	*assimilis*	*inermis*	*javanus*	*liuae*	*meruensis*	*simplex*
		[12,44,55]	[12,55]	[56]	[57]	[12,44,58]	[56,59]
	Type Locality	Debundscha,	Debundscha,	Bogor, West	Shitai,	Meru,	Bukit Timah,
		Cameroon	Cameroon	Java, Indonesia	China	Kenya	Singapore
Characteristics	Species Group	*gracilis*	*gracilis*	*sundanensis*	*sundanensis*	*gracilis*	*sundanensis*
Body bluish/violetish		Entire body, except	Ant., legs and	Ant. only	Ant., femurs and	Entire body or	Ant., legs and
pigmentation on		dorsal head and trunk	Abd. V–VI		tibiotarsi	just Ant. and legs	ventral body
		and ventral dens					
Dorsal head mac	**S2**	‒	‒	+	+	‒	+
	**Pa1**	‒	‒	+	+	‒	‒
	**Pp3**	+	‒	+	+	+	+
	**Pp5**	+	‒	+	+	‒	+
Labial basomedian field	**M1**	?	?	S	C	C	?
	**M2**	?	?	S	S	S	?
	**R**	?	?	C	S	S	?
	extra chaetae	?	?	3 C	4 S	6 C	?
Th. II mac	**m4** group	3	2	2	2	2	2
	**p1–3** group	6	7	6	8	6	6–7
	**p5** mac	‒	‒	+	+	‒	‒
Th. III central mac	**a, m, p** rows	4,2,3	4,2,3	5,1,3	5,1,3–4	4,2,3	4,0,3
Trochanteral organ chaetae		?	?	50	30	?	70
Tibiotarsus III	inner	+?	+?	‒	+?	+?	?
smooth chaetae	outer	‒	+?	‒	‒?	‒	?
Tenent hair		acuminate	capitate	acuminate	acuminate	acuminate	acuminate
Unguis teeth	ratio	b.t. > m.t.	b.t. = m.t.	b.t. > m.t.	b.t. = m.t.	b.t.=m.t.	b.t. only *
	**m.t.**	+	+	+	+	+	‒?
Unguiculus lamellae	**ai**	acuminate	truncate, 1 tooth	acuminate	acuminate	acuminate	acuminate
	**pe**	1 tooth	1 tooth	smooth	smooth	1 tooth	smooth
Tenaculum chaetae		?	?	2	2–4	?	?
Manubrium dorsal	number	?	?	6	+?	+?	3
smooth chaeta	distribution	?	?	2 rows	+?	+?	2 rows
Dens (basal) smooth chaeta		?	?	1	5	+?	2
Dens (dorsal) blunt mac		‒	‒	+	‒	‒	‒
Dental spines	number	‒	‒	‒	‒	±40–46	5–8
	distribution	‒	‒	‒	‒	3–4 rows	1 row

Legends: [] references; (+) present; (‒) absent; (±) approximately; (?) unknown/unclear; (S) smooth; (C) ciliate; ungual teeth = (b.t.) basal paired teeth, (m.t.) medial unpaired tooth; unguiculus lamellae = (ai) antero-internal lamella, (pe) postero-external lamella. * Yosii’s drawing [59, pg. 41, figure 24D] and his text description point to the presence of only the medial tooth, however it is more likely it is the basal pair of minute teeth more distally displaced.

A key to the Brazilian species of *Dicranocentrus* is presented below.

Identification key of Brazilian *Dicranocentrus* species *^,^**

1. Head post-occipital series with 0–1 (**Pa5**) mac (Figure 2F) … (*marias* group) 2

– Head post-occipital series with 2 or more mac … (*gracilis* group) 4

2. Th. II with 5–6 posterior mac; Th. III with 9 central mac; Abd. I with 5 mac … *D. pikachu* Xisto and Mendonça, 2017

– Th. II with 4 posterior mac; Th. III with 6 central mac; Abd. I with 3 or less mac (Figure 4A–C) … 3

3. Abd. I–II with 3 and 2 central mac, respectively (Figure 4C–D); Abd. III with 1 lateral mac; Abd. IV with 4–5 central mac (Figure 5A–B); unguiculi leaf shaped and wide, with one posterior tooth (Figure 6B) … *D. abestado* sp. nov. 

– Abd. I – II with 2 and 1 central mac, respectively; Abd. III with 2 lateral mac; Abd. IV with 3 central mac; unguiculi normal without posterior teeth … *D. amazonicus* Bellini, Moraes and Oliveira, 2013

4. Head post-occipital series with 2 mac … 5

- Head post-occipital series with 4–5 mac … 6

5. Trunk pigmented; head **An** row with 14 mac; labial basomedian field with **m1** smooth and devoid of extra chaetae and scales; Th. II with 6 posterior mac; unguis apical tooth absent … *D. cuprum* Xisto and Mendonça, 2016

– Trunk depigmented; head **An** row with 12 mac; labial basomedian field with **M1** ciliate, 1 extra smooth chaeta and scales present; Th. II with 7 posterior mac; unguis apical tooth present … *D. heloisae* Arlé and Mendonça, 1982

6. Head post-occipital series with 4 mac; unguis apical tooth present … 7

– Head post-occipital series with 5 mac; unguis apical tooth absent … 8

7. Th. III to Abd. V and tibiotarsi distally pigmented; head **An** row with 15 mac; labial basomedian field with **R** and 1 extra chaeta ciliate; Th. II with 6 posterior mac; trochanteral organ with about 60 spine-like chaetae; dens with about 110 spines … *D. marimutti* Xisto and Mendonça, 2017

– Head, trunk and coxae to most of tibiotarsi pigmented; head **An** row with 13 mac; labial basomedian field with **r** and 1 extra chaeta smooth; Th. II with 7 posterior mac; trochanteral organ with about 100 spine-like chaetae; dens with about 60 spines … *D. magnus* Xisto and Mendonça, 2017

8. Trunk and manubrium heavily pigmented; head with **S6i** mac and 3 interocular chaetae; maxillary outer lobe basal chaeta blunt; labial basomedian field with **R** ciliate and devoid of extra chaetae; Abd. III with 1 lateral mac … *D. albicephalus* Xisto and Mendonça, 2017

– Pigments restricted to antennae and legs (weakly); head devoid of **S6i** mac, with 4 interocular chaetae; maxillary outer lobe basal chaeta acuminate; labial basomedian field with **r** smooth and 2 extra chaeta (one smooth, the other ciliate); Abd. III with 2 lateral mac … *D. melinus* Xisto and Mendonça, 2016

Notes: (*) *D. bicolor* Handschin, 1924, *D. termitophilus*, Handschin 1924 and *D. silvestrii* Absolon, 1903 were not included since they were considered *species inquirendae* (see the previous topic); (**) All Brazilian species of *Dicranocentrus* are only known from Brazil, with the exception of *D. silvestrii*.

### 4.2. Notes on Dicranoncentrus Chaetotaxy

*Dicranocentrus* species morphology is widely variable as partially represented in Table 2, Table 3, Table 4 and Table 5. Dorsal head macrochaetotaxy can have, among other chaetae, **S2**, **S6i** and several posterior mac (on **Pa**, **Pm** and **Pp** rows) or can be devoid of at least some of these mac [12]. Trunk dorsal macrochaetotaxy of most species has few or none secondary mac, other than those on the Th. II anterior collar, **m4** and **p** groups, and **m3e** on Abd. II, but differences in the presence and number of some primary mac or their multiples occur. Such data are of taxonomic relevance, especially on Th. II–III and Abd. I [12]. The number and displacement of smooth acuminate chaetae on proximal antennae, tibiotarsi, manubrium and proximal dens are of specific value, as well as the presence, number and position of dental spines. Labial basomedian field **M** and **R** chaetae multiples, which can include scales on some species, are so inconstant and can be so abundant that in some cases can prevent a clear understanding of which are some of the primary chaetae. Even the tenaculum corpus chaetotaxy diverges among the species (and can be polymorphic in few ones), as represented in Table 4 and Table 5. Such variations among the species plus our finding of *D. abestado* sp. nov. with an unexpected number of seven sens on Abd. V (previously known as only four, but unknown to most taxa) may point out to an artificial status for *Dicranocentrus*. For instance, *Sinodicranocentrus* Zhang, 2020 [15] was recently erected based especially on ungual morphology and Abd. V S-chaetotaxy, and it was supported by molecular data; otherwise *Dicranocentrus* would be paraphyletic ([15], p. 15, Figure 1). In this sense it is possible that the widely variable morphology of *Dicranocentrus* may hide more distinct lineages of Heteromurini, and further studies on its morphology and systematics may unveil this condition. This approach could also confirm/refute Mari-Mutt’s groups of species [12].

### 4.3. Notes on Dicranocentrus Species Groups and Related Genera

Mari-Mutt’s groups of species based on dorsal head macrochaetatoxy are quite useful for the current taxonomy of *Dicranocentrus* [12,17,18,20,21,41,57]. Distribution endorses Mari-Mutt’s groups at some level [12] (as partially represented in Figure 7). For instance, the *marias* group is almost exclusively Neotropical, with the exception of *D. spinosus* Prabhoo, 1971 [68] described from India. This group is well delimited by the reduction in dorsal head macrochaetotaxy, devoid of **S2** and most post-occipital mac, aside from **Pa5** (which can be rarely absent as well) and **Pp5** (only seen in *D. paramoensis*) [12].

The *gracilis* and *sundanensis* groups are characterized by a more complex dorsal head macrochaetotaxy, with post-occipital series with two or more mac, but they differ from each other by the presence (*sundanensis*) or absence (*gracilis*) of **S2** mac. Other morphological features of both are mixed and should be better investigated since they raise doubts if the *gracilis* and *sundanensis* groups are really independent taxa. For example, both groups hold species with one or two central mac on Abd. III, with or without dental spines, and with or without a very peculiar proximal blunt mac on dorsal dens, described to South Asian species as *D. janetscheki* Yosii, 1971 [69], *D. indicus* and *D. javanus*, from the *sundanensis* group, and *D. fraternus* Mari-Mutt and Bhattacharjee, 1980 [53], from the *gracilis* group [53,56,60,69]. The head chaetotaxy of *D. janetscheki* and *D. indicus* are unknown, and thus it is not entirely clear if they really belong to the *sundanensis* group [12,69]; *D. javanus*, however, belong to that group [56].

The presence of 1 proximal dental mac was also reported to *Falcomurus chilikaensis* Mandal, 2018 [70] (Heteromurini), the sole species of the genus described from India. This species was compared specially with *Heteromurus* due to the presence of only Ant. I subdivided (so, with five antennal segments) and Abd. I devoid of mac [70]. However, there are incongruences on this species description [15], which may point to an inaccurate interpretation of Abd. I chaetotaxy as well. Additionally, *Dicranocentrus* specimens can rarely have four or five antennal segments, as juveniles or when the antennae are regenerating [12]. Finally, among the Orchesellidae, only species of *Dicranocentrus* and now *Falcomurus* have a proximal dental mac. Because of this, it is possible that *Falcomurus* represents a *Dicranocentrus* species.

Mari-Mutt’s phylogeny suggested the *marias* and *gracilis* groups had a close ancestral link [12]. Due to the wide Pantropical distribution of the *gracilis* group [3,12], it is possible it was already established at least during the Jurassic (about 180 mya), and the *marias* group emerged from a *gracilis*-like ancestor after the brake of western Gondwana in the Neotropical Region. In this sense, the presence of *D. spinosus* of the *marias* group in India was possibly due to: (1) human-mediated dispersal events, such as plant and soil carrying during the past five centuries; (2) it emerged independently from the Neotropical *marias* group, and its resemblance with other taxa is due to convergence. Mari-Mutt’s phylogeny also suggested that the *sundanensis* group and *Pseudodicranocentrus* Mari-Mutt, 1981 [45] (formerly *Dicranocentrus circulatus* group) are closely related [12]. The distribution of the *sundanensis* group is Oriental to Australasian (as partially represented in Figure 7), while *Pseudodicranocentrus* is endemic to Mexico and Guatemala [3,9,45]. If Mari-Mutt’s hypothesis is correct, the presence of *Pseudodicranocentrus* in Central America possibly occurred by the dispersal of its ancestor by the Pacific Ocean, similarly to the model of Christiansen and Bellinger for the Hawaiin colonization [71].

Below, we provide an identification key to all species of *Dicranocentrus* with 3 mac on Abd. I. They belong to *marias*, *sundanensis* and *gracilis* groups. Further data on such species are presented in the remarks on the new species and in Table 2, Table 4 and Table 5.

Identification key of *Dicranocentrus* species with 3 mac on Abd. I

1. Dorsal dens with 1 proximal blunt mac … 2

– . Dorsal dens devoid of blunt mac … 3

2. Th. II with 8 posterior mac, **p5** mac absent; Abd. III with 2 central mac; trochanteral organ with about 29 spine-like chaetae; tenent hairs capitate; tenaculum with 1 chaeta; dorsal manubrium devoid of smooth chaetae … *D. indicus* Bonet, 1930*

– Th. II with 7 posterior mac, **p5** mac present; Abd. III with 1 central mac; trochanteral organ with about 50 spine-like chaetae; tenent hairs acuminate; tenaculum with 2 chaetae; dorsal manubrium with 6 smooth chaetae in 2 rows … *D. javanus* Yoshii and Suhardjono, 1989

3. Head post-occipital series with 0–1 (**Pa5**) mac (Figure 2F); Neotropical distribution … (*marias* group) 4

– Head post-occipital series with 2 or more mac; Old World distribution (Figure 7) … 8

4. Labial basomedian field devoid of R chaeta; Th. III with 8 central mac; Abd. IV devoid of **B5** mac … *D. antillensis* Mari-Mutt, 1979

– Labial basomedian field with **R** chaeta; Th. III with 6 central mac; Abd. IV with **B5** mac … 5

5. Labial basomedian field with 6–7 extra ciliate chaetae; Th. II **m4** group with 2 mac, with 5 posterior mac; Th. III a, m, p rows with 2, 1, 3 mac respectively … *D. paramoensis* Mari-Mutt, 1983

– Labial basomedian field with 1–3 extra ciliate chaetae; Th. II **m4** group with 3 mac, with 4 posterior mac; Th. III **a**, **m**, **p** rows with 3, 0, 3 mac respectively … 6

6. Labial basomedian field **R** chaeta ciliate; ungues with apical tooth; unguiculi leaf shaped and wide … *D. abestado* sp. nov. 

– Labial basomedian field **r** chaeta smooth; ungues devoid of apical tooth; unguiculi normal … 7

7. Labial basomedian field with 3–4 extra smooth chaetae; unguiculi devoid of outer teeth … *D. icelosmarias* Soto-Adames and Anderson, 2017

– Labial basomedian field with 1–3 extra smooth chaetae; unguiculi with 1 outer tooth … *D. marias* Wray, 1953

8. Head sutural series with 7 mac (**S2** absent, Figure 2F) … (*gracilis* group) 9

– Head sutural series with 8 mac (**S2** present) … (*sundanensis* group) 12

9. Head post-occipital series with 10 mac; Th. III **a**, **m**, **p** series with 5, 1, 3 mac, respectively; Abd. III with 2 central mac (Figure 5A) … *D. nepalensis* Mari-Mutt, 1980

– Head post-occipital series with 6 or less mac; Th. III **a**, **m**, **p** series with 4, 2, 3 mac, respectively; Abd. III with 1 central mac … 10

10. Dental spines present … *D. meruensis* Wahlgren, 1908

– Dental spines absent … 11

11. Head with **Pp3** and **Pp5** mac; Th. II **m4** group with 3 mac, with 6 posterior mac; tenent hairs acuminate; unguiculi acuminate without inner teeth … *D. assimilis* Schött, 1927

– Head devoid of **Pp3** and **Pp5** mac; Th. II **m4** group with 2 mac, with 7 posterior mac; tenent hairs capitate; unguiculi truncate with 1 inner tooth … *D. inermis* Schött, 1927

12. Abd. III with 1 central mac … 13

– Abd. III with 2 central mac (Figure 5A) … 14

13. Head with **Pa1** mac; Th. II with 9 posterior mac, **p5** mac present; Th. III **a**, **m**, **p** series with 5, 1, 3–4 mac, respectively; trochanteral organ with about 30 spine-like chaetae; dental spines absent … *D. liuae* Xu and Zhang, 2014

– Head devoid of **Pa1** mac; Th. II with 6–7 posterior mac, **p5** mac absent; Th. III **a**, **m**, **p** series with 4, 0, 3 mac, respectively; trochanteral organ with about 70 spine-like chaetae; dental spines present … *D. simplex* Yosii, 1959

14. Dental spines present … 15

– Dental spines absent … 16

15. Dorsal head **Pp3** mac absent; Th. II devoid of **p5** mac; Th. III with 9 central mac; Abd. IV with 3 central mac; dorsal manubrium with 5–6 smooth chaetae; dens with 14–20 spines … *D. luzonensis* Mari-Mutt, 1985

– Dorsal head **Pp3** mac present; Th. II with **p5** mac; Th. III with 12 central mac; Abd. IV with 4–5 central mac; dorsal manubrium with 23–25 smooth chaetae; dens with 8–13 spines … *D. wangi* Ma and Chen, 2017

16. Labial basomedian field chaetae **M1–2** ciliate; Th. II **p1–3** group with 7 mac, **p5** mac present; Abd. IV with 3 central mac; manubrium devoid of smooth chaetae … *D. inermodentes* (Uchida, 1944)

– Labial basomedian field chaetae **m1**–**2** smooth; Th. II **p1–3** group with 8 mac, **p5** mac absent; Abd. IV with 4–5 central mac; dorsal manubrium with 2 rows of smooth chaetae … 17

17. Labral papillae hooked; labial basomedian field with 2–3 extra smooth chaetae; Th. III with 9 central mac; dorsal manubrium with 3–4 smooth chaetae on each row … *D. gemellus* Mari-Mutt, 1985

– Labral papillae conical; labial basomedian field with 4–6 extra smooth chaetae; Th. III with 11 central mac; dorsal manubrium with 5 smooth chaetae on each row … *D. solomonensis* Mari-Mutt, 1979

Notes: (*) The head chaetotaxy of *D. indicus* is unknown (Table 4). Nevertheless, Mari-Mutt [12] suggests it is from the *sundanensis* group, closely related to *D. inermodentes*. Ma and Chen [54] key considered the **S2** mac present in *D. indicus,* possibly based on [12], but apparently no material was examined. Because of this we considered such information as dubious.

### 4.4. Notes on Heteromurinae Systematics

Although the internal evolution of Heteromurinae was, to some extent, recently studied [15], its affinities with other Entomobryoidea are not entirely clear [2,72]. Recent advances in Entomobryoidea/Entomobryidae systematics support both a closer relationship of Heteromurinae with derived taxa as the Entomobryinae, Seirinae, Lepidocyrtinae and Paronellidae [26,72,73,74], or with other Orchesellidae [2,75]. For now, Heteromurinae is grouped with unscaled Orchesellidae [2], but the morphology of this subfamily, with an overall reduction in trunk dorsal mac multiples, several primary chaetae as mic, the presence of coarsely ciliate scales as secondary coverage and reduced S-chaetotaxy formula (2, 2|1, 3, 3 on Th. II to Abd. III) suggest that this taxon is possibly more derived than most unscaled Orchesellidae [12,40,57,72]. Even though Heteromurinae and other Orchesellidae share a short Abd. IV and a postantennal organ (seen only in *Alloscopus* among the Heteromurinae, variable among other Orchesellidae), such features should be interpreted as plesiomorphies, as well as the presence of hook-like labral papillae and tenaculum with more than one chaeta, also seen in some of their species. In this context, if we consider the alternative hypothesis to Entomobryoidea topology as Orchesellidae + (Heteromurinae + Entomobryidae/Paronellidae), the presence of scales could be the synapomorphy of the more derived Entomobryoidea, including Heteromurinae, and it was secondarily lost possibly more than one time among the Entomobryinae [75,76]. To support this interpretation, the basal Entomobryinae and all Seirinae also share coarsely ciliate scales with the Heteromurinae [72,75,77].

## 5. Conclusions

After the description of *Dicranocentrus abestado* sp. nov. there are now 12 species of the genus recorded from Brazil. Its Abd. V S-chaetotaxy with seven sens, unmatched in the genus, combined with the widely variable morphology of *Dicranocentrus* species, and the recent description of *Sinodicranocentrus* based on ungual morphology and S-chaetotaxy raises doubts about the validity of the former genus. Regarding the Brazilian *species inquirendae*, there is arguably enough data for the collection of fresh specimens of *D. bicolor* and *D. termitophilus* and their redescription. Conversely, *D. silvestrii* identity, including its real type locality, remains obscure and such a name cannot be used with certainty. The Heteromurinae position within Entomobryoidea remains puzzling, but the morphology, especially the presence of scales, may indicate its affinities with more derived taxa than the unscaled Orchesellidae.

## Figures and Tables

**Table 1:** List of Brazilian species of Orchesellidae Börner, 1906 *sensu* Zhang et al. [2] and their known distribution.

Taxa	Known Distribution in Brazil (Biomes)
**Heteromurinae Absolon and Ksenemann, 1942** [22]	
**Heteromurini Absolon and Ksenemann, 1942** [22]	
*Heteromurtrella* Mari-Mutt, 1979 [23]	
*H. anae* Cipola, 2016 * [14]	Amazon Forest
*Dicranocentrus* Schött, 1893 [7]	
*D. albicephalus* Xisto and Mendonça, 2017 * [21]	Atlantic Forest
*D. amazonicus* Bellini, Moraes and Oliveira, 2013 * [17]	Amazon Forest
*D. bicolor* Handschin, 1924 *^,^*** [13]	Atlantic Forest
*D. cuprum* Xisto and Mendonça, 2016 * [20]	Atlantic Forest
*D. heloisae* Arlé and Mendonça, 1982 * [16]	Atlantic Forest
*D. magnus* Xisto and Mendonça, 2017 * [21]	Atlantic Forest
*D. marimutti* Xisto and Mendonça, 2017 * [21]	Atlantic Forest
*D. melinus* Xisto and Mendonça, 2016 * [20]	Atlantic Forest
*D. pikachu* Xisto and Mendonça, 2017 * [21]	Atlantic Forest
*D. silvestrii* Absolon, 1903 **^,^*** [24]	Atlantic Forest
*D. termitophilus* Handschin, 1924 *^,^*** [13]	Atlantic Forest?
**Mastigocerini Mari-Mutt, 1980** [25]	
*Mastigoceras* Handschin, 1924 * [13]	
*M. camponoti* Handschin, 1924 *^,^** [13]	Atlantic Forest, Amazon Forest, Caatinga
**Nothobryinae Zhang and Deharveng, 2015** [26]	
**Nothobryini Soto-Adames et al., 2008** [27]***sensu* Nunes et al. 2020** [28]	
*Nothobrya* Arlé, 1961 * [29]	
*N. arlei* Silveira and Mendonça, 2016 *[30]	Atlantic Forest
*N. schubarti* Arlé, 1961 * [29]	Cerrado, Caatinga
*N. sertaneja* Nunes and Bellini, 2019 * [31]	Caatinga
**Capbryini Nunes et al. 2020** [28]	
*Capbrya* Barra, 1999 [32]	
*Capbrya brasiliensis* Nunes, Santos-Costa and Bellini, 2020 * [28]	Caatinga, Cerrado, Atlantic Forest

Legends: [] description references; (*) endemic from Brazil; (**) possibly a species complex; (***) *Species inquirenda* (see the discussion topic); (?) unclear data.

**Table 2:** Comparison among *Dicranocentrus* species of the *marias* group from the New Word with similar morphology (head **S** series with 7 mac, basomedian labial field with **M1** chaeta ciliate, **m2** and **e** smooth, papilla **E** lateral process short, Th. II without **p5** mac and Abd. I–III with 3, 2 and 2 central mac, respectively).

	Species [References]	*abestado*	*antillensis*	*icelosmarias*	*marias*	*paramoensis*
		sp. nov.	[12,45]	[48]	[12,45,49]	[47]
	Type Locality:	Chapada Diamantina	Camp Perrin,	Brick Kiln,	Las Marias,	Páramo de Mucubají,
Characteristics		Bahia, Brazil	Haiti	Saint Kitts and Nevis	Puerto Rico	Mérida, Venezuela
Body bluish/violetish		Ant., anterior head, lateral Th., dorsal Abd. VI and part of legs	Ant., anterior head, part of legs, lateral Abd. I–IV and posterior Abd. III–IV		Ant., anterior head, lateral trunk, legs or entirely pigmented	Entire body and appendages, except furca
Pigmentation on:				Unpigmented **		
	
Interocular chaetae		5	?	3–4	?	?
Dorsal head mac	**Pp5**	−	−	−?	−	+
Basomedian and	**R** (smaller)	C	−	S	S	S/C
basolateral labial fields	**L1**	S	?	S	?	C
	**L2**	S	?	S	?	C
	extra chaetae *	0–1 S, 2–3 C	−	3–4 S, 1C	1–3 S, 1–2 C	0–1 S, 6–7 C
	scales	−	+	?	−	−
Cephalic groove b.c.		3	?	3	?	2
Th. II mac	**m4** group	3	2	3	3	2
	**p** row	4	5	4	4	5
Th. III central mac	**a, m, p** rows	3,0,3	4,1,3	3,0,3	3,0,3	2,1,3
Abd. IV central mac	**B5**	+	−	+	+	+
	**B6**	+/−	+	+	+/−	−
Trochanteral organ chaetae		22–27	?	±23	?	35–52
Tibiotarsus III inner smooth chaetae		5	−	+	+	6

Tenent hair		acuminate/capitate	acuminate/capitate	capitate	acuminate/capitate	acuminate
Unguis teeth	ratio	b.t.=m.t. > a.t.	b.t. = m.t.	b.t. > m.t..	b.t. > m.t..	b.t. > m.t. > a.t.
	**a.t.**	+	−	–	−	+/−
Unguiculus lamellae	**ai**	wide	normal	normal	normal	normal
	**pe**	1 tooth	1 tooth	smooth	1 tooth	1 tooth
Manubrium dorsal smooth mac		3 + 3	−	4 + 4	?	4 + 4

Dental spines	number	16–20	?	20	16–42	±16
	distribution	2–3 rows grouped	variable	2 rows grouped	2–5 rows	2 rows grouped

Legends: [] references; (+) present; (−) absent; (±) approximately; (/) or; (?) unknown/unclear; (S) smooth; (C) ciliate; (b.c.) basal chaetae; ungual teeth = (b.t.) basal paired teeth, (m.t.) medial unpaired tooth, (a.p.) apical unpaired tooth; unguiculus lamellae = (ai) antero-internal lamella, (pe) postero-external lamella; (*) extra chaetae only on labial basomedian field; (**) Soto-Adames and Anderson [48] only described the background colour, devoid of blue pigments. Distal antennae colour unknown for this species.

**Table 3:** Comparison among the Brazilian *Dicranocentrus* species.

	Species [References]	*abestado* sp. nov.	*albicephalus* [21]	*amazonicus* [17]	*bicolor* [13]	*cuprum* [20]	*heloisae* [16,18]	*magnus* [21]	*marimutti* [21]	*melinus* [20]	*pikachu* [21]	*termitophilus* [13]
	Type Locality	Chapada Diamantina, Bahia	Serra dos Órgãos, Rio de Janeiro	Reserva Ducke, Amazonas	Blumenau, Santa Catarina	Serra da Gandarela, Minas Gerais	Floresta da Tijuca, Rio de Janeiro	Serra do Japi, São Paulo	Serra dos Órgãos, Rio de Janeiro	Serra da Gandarela, Minas Gerais	Serra do Japi, São Paulo	Minas Gerais
Characteristics	Species Group	*marias*	*gracilis*	*marias*	?	*gracilis*	*gracilis*	*gracilis*	*gracilis*	*gracilis*	*marias*	?
Body bluish/violetish pigmentation on:	Ant.	+	+	+	Ant. II–IV	partially	+	partially	Ant. II–IV	+	partially	mostly
	Head (– eyes)	anteriorly	anteriorly	weakly	−	+	+	partially	−	anteriorly	−	anteriorly
	Th. (dorsally)	−	+	−	−	+	−	+	Th. III	−	−	partially
	Abd. (dorsally)	Abd. VI	+	−	−	+	−	partially	+	−	−	partially
	Legs	partially	partially	weakly	mostly	proximally	−/+	mostly	tibiotarsi	+	tibiotarsi	–?
Ant. IIb smooth mac		3	−?	?	?	−?	6?	1?	4?	−?	−?	?
Interocular chaetae		5	3	3	?	3	3	3	4	4	3	?
Dorsal head mac	**An** row	11–12	14–15	±11	?	14	12	13	15	13	17	?
	**S6i**	+	+	+	?	+	+	+	+	−	+	?
	**posterior**	1	5	1 *	?	2	2	4	4	5	1	?
Maxillary outer lobe b.c.		acuminate	blunt	acuminate	?	acuminate	acuminate	acuminate	acuminate	acuminate	blunt	?
Labial basomedian field	**M1**	C	C	C	?	S	C	C	C	C	C	?
	**R** (smaller)	C	C	S	?	S	S	S	C	S	C	?
	extra chaetae	0–1 S, 2–3 C	−	2 S, 1 C	?	−	1 S	1 S	1 C ***	1 S, 1 C	1 C	?
	scales	−	−	−	?	−	+	−	−	−	−	?
Th. II mac	**p** row	4	6	4	?	6	7	7	6	6	5–6	?
Th. III central mac	**a,m,p** rows	3,0,3	5,1,3	3,0,3	?	5,1,3	5,1,3	5,1,3	5,1,3	5,1,3	5,1,3	?
Abd. I mac		3	5	2	?	5	5	5	5	5	5	?
Abd. II mac	central	2	2	1	?	2	2	2	2	2	2	?
Abd. III mac	lateral	1	1	2	?	2	2	2	2	2	2	?
Abd. IV central mac		4–5	5	3	?	5	5	5	5	5	5	?
Trochanteral organ chaetae		22–27	±40	±30	?	±70	±100	±100	±60	±65	±80	?
Tenent hair		acuminate /capitate	acuminate	capitate	capitate	acuminate	acuminate/capitate	acuminate	acuminate	capitate	capitate	acuminate
Unguis teeth	ratio	b.t. = m.t.>a.t.	b.t. = m.t.	b.t. = m.t.	b.t. = m.t.	b.t. = m.t.	b.t. = m.t.>a.t.	b.t. = m.t.>a.t.	b.t. = m.t. > a.t.	b.t. = m.t.	b.t. < m.t.	b.t. = m.t.
	a.t.	+	−	−	−	−	+	+	+	−	−	−
Unguiculus lamellae	ai	wide	normal	normal	normal	normal	normal	normal	narrow	normal	narrow	normal
	pe	1 tooth	1 tooth	S	1 tooth	1 tooth	1 tooth	1 tooth	1 tooth	1 tooth **	1 tooth	S
Dental spines	number	16–20	±50	±19	±30?	±50	±60	±60	±110	±55	±50	20–30
	distribution	2–3 rows grouped	5–6 rows grouped	2 rows grouped	4 rows grouped	grouped	7 rows	6–7 rows	variable	mostly grouped	grouped	3 rows grouped

Legends: [] references; (+) present; (−) absent; (±) approximately; (/) or; (?) unknown/unclear; (S) smooth; (C) ciliate; (b.c.) basal chaetae; ungual teeth = (b.t.) basal paired teeth, (m.t.) medial unpaired tooth, (a.p.) apical unpaired tooth; unguiculus lamellae = (ai) antero-internal lamella, (pe) postero-external lamella; (*) not represented in Bellini et al. [17]; (**) represented on antero-external lamella lamella; (***) **a1** not represented [21, pg 30, figure 15i]. *D. silvestrii* Absolon, 1903 was not included, see the discussion topic.
